# Long-Term Outcomes of Transobturator Mid-Urethral Sling for the Treatment of Stress Urinary Incontinence Among Chinese Women

**DOI:** 10.1007/s00192-026-06605-8

**Published:** 2026-03-21

**Authors:** Mei Chi Cheng, Yuk Sheung Fan, Willy Cecilia Cheon

**Affiliations:** Department of Obstetrics and Gynaecology, Queen Mary Hospital, Hong Kong, Hong Kong

**Keywords:** Chinese women, Long-term follow-up, Mid-urethral sling, Stress urinary incontinence, Tension-free vaginal tape obturator, TVT-O

## Abstract

**Introduction and Hypothesis:**

Transobturator mid-urethral sling (TVT-O), a surgical treatment for stress urinary incontinence, has been shown to be highly effective in the short and medium term. However, long-term outcomes with larger sample sizes are lacking. The study is aimed at assessing the efficacy and safety of TVT-O over a 10-year follow-up period in Chinese women.

**Methods:**

This prospective observational study included Chinese women who underwent TVT-O for urodynamic stress incontinence. Objective cure was defined as the absence of urodynamic stress incontinence on a urodynamic study, whereas a subjective cure was defined as the patient’s perception of their condition. Quality of life (QOL) was assessed using the validated Urogenital Distress Inventory-6 questionnaire and the Incontinence Impact Questionnaire-7, along with the visual analogue scale (VAS).

**Results:**

A total of 326 Chinese women were included in the study. The objective and subjective cure rates at the 10-year follow-up were 75.2% and 73.6% respectively. Significant improvements were observed across all QOL domains. The mean VAS satisfaction score was 8.5 out of 10 after 10 years. Complications were uncommon; 2 women (0.6%) experienced tape erosion, one of whom required local excision. No patients reported persistent thigh pain after 10 years. The cumulative rate of repeating the mid-urethral sling procedure was 2.5%.

**Conclusions:**

The TVT-O is a highly effective and safe surgical treatment for women with stress urinary incontinence at the 10-year follow-up. Thigh pain after TVT-O is rare and should not prevent women from considering this beneficial treatment.

## Introduction

Stress urinary incontinence (SUI) is defined as involuntary leakage of urine upon effort or exertion or upon sneezing or coughing [1]. SUI is a common problem in Hong Kong. According to a territory-wide telephone survey conducted in Hong Kong published in 2006, 40.8% and 15.9% of women reported SUI and mixed urinary incontinence respectively [2]. In addition, this survey revealed that 16.0% of the women who had urinary incontinence reported quality-of-life impairment; 9.3% felt frustrated with low morale, and 15.2% were nervous and anxious.

In the latest published Population Census in Hong Kong in 2021 [3], one-fifth of the population was aged 65 or above, and this percentage has been increasing over the past 30 years. However, the sex ratio (number of males per 1000 females) has been decreasing, suggesting that there are more females than males in Hong Kong. SUI is becoming more prevalent as the population ages, and the life expectancy of women in Hong Kong is one of the longest in the world. The burden of SUI is expected to increase. Therefore, effective and safe surgery for SUI is crucial for maintaining a good quality of life (QOL) in these women.

Mid-urethral sling operations, including transobturator and retropubic routes, are highly effective in the short and medium term as a surgical treatment for SUI in women [4]. Long-term (up to 17 years) favourable outcomes in 70 patients who underwent transobturator mid-urethral sling (TVT-O) surgery have recently been published [5]. Mid-urethral sling operations have good safety profiles. The transobturator route is associated with a lower rate of bladder perforation than the retropubic route, but a possibly a higher rate of groin pain [4].

The aim of this prospective study was to assess the long-term efficacy of TVT-O for the treatment of SUI in a large sample of Chinese women in Hong Kong. The safety profile of this surgery was also evaluated.

## Materials and Methods

This prospective observational study included women who were undergoing TVT-O procedures between 2003 and 2015. All women were recruited from the Department of Obstetrics and Gynaecology of a tertiary hospital in Hong Kong, which is also a urogynaecology subspecialty training centre.

The inclusion criteria included women who were Chinese, older than 18 years, mentally fit for written consent, and diagnosed with urodynamic stress incontinence (USI) via a urodynamic study (UDS) and those who had a TVT-O inserted for USI and were capable of physical urodynamic assessment. Women who were non-Chinese and who refused or were unable to provide written consent, and who could not understand either Chinese or English, had cognitive impairment or were a psychiatric patient, had a history of tape excision or tape removal for any reason upon recruitment or had symptoms of urinary tract infection upon recruitment were excluded.

Clinical assessments and UDS were performed on women who presented with SUI. Any cases of concomitant pelvic organ prolapse (POP) that were staged using the Pelvic Organ Prolapse Quantification system were recorded, and surgery for the correction of POP was discussed as appropriate. All UDS were conducted using a standardized protocol according to the Good Urodynamic Practice guidelines of the International Continence Society [5]. A pad test was also performed at the time of the UDS.

All procedures were performed or supervised by urogynaecologists. Five urogynaecology subspecialty trainees were under training during the study period. Prolapse surgery or other concomitant surgeries were performed first. A TVT-O was then inserted. The bladder was catheterized. A longitudinal incision to the vaginal mucosa was made at mid-urethral level. The paravaginal space was dissected towards the medial border of the inferior pubic rami. Incisions were made at the bilateral groin region. A metal guide was put into the dissected tract of the paravaginal space. TVT-O needles were inserted via the vaginal incision along the metal guide, through the obturator membranes and went out via the groin incisions. The vaginal wall was checked for integrity. Cystoscopy was performed with the tape in situ to check for any bladder or urethral injury. The tension of the tape was then adjusted. The vaginal wound was closed with polyglactin sutures.

After TVT-O with or without concomitant surgery, the clinical assessments, UDS test, and pad test were performed on the recruited women at postoperative years 1, 5 and 10. The presence or absence of postoperative thigh or groin pain was particularly questioned at the 3-month and 10-year postoperative follow-up visits.

Quality-of-life assessments, using the validated Urogenital Distress Inventory-6 (UDI-6) questionnaire and the Incontinence Impact Questionnaire-7 (IIQ-7) [6], were performed preoperatively and at 1, 5 and 10 years after surgery. Visual analogue scale (VAS) scores (0–10, with 10 being most satisfied) were also assessed 10 years after surgery for subjective satisfaction regarding SUI after surgery.

An objective cure was defined as the absence of USI on a UDS, whereas a subjective cure was defined as the patient’s perception of their condition.

The primary outcomes of this study were the objective and subjective cure rates at 1, 5 and 10 years postoperatively. Secondary outcomes included any thigh or groin pain at 3 months and 10 years postoperatively, the pad test results, UDI-6 and IIQ-7 questionnaire scores and VAS scores. Subgroup analysis was also performed on the objective and subjective cure rates at 10 years postoperatively between women who had TVT-O alone and women who had TVT-O plus concomitant surgery.

Statistical analysis was performed using Statistical Package for the Social Sciences software, version 13. The McNemar test was used to compare the preoperative and postoperative parameters. Statistical significance was defined as *p* < 0.05.

This study was conducted in accordance with the Declaration of Helsinki and was approved by the Central Institutional Review Board of Hospital Authority in Hong Kong.

## Results

**Fig. 1:**
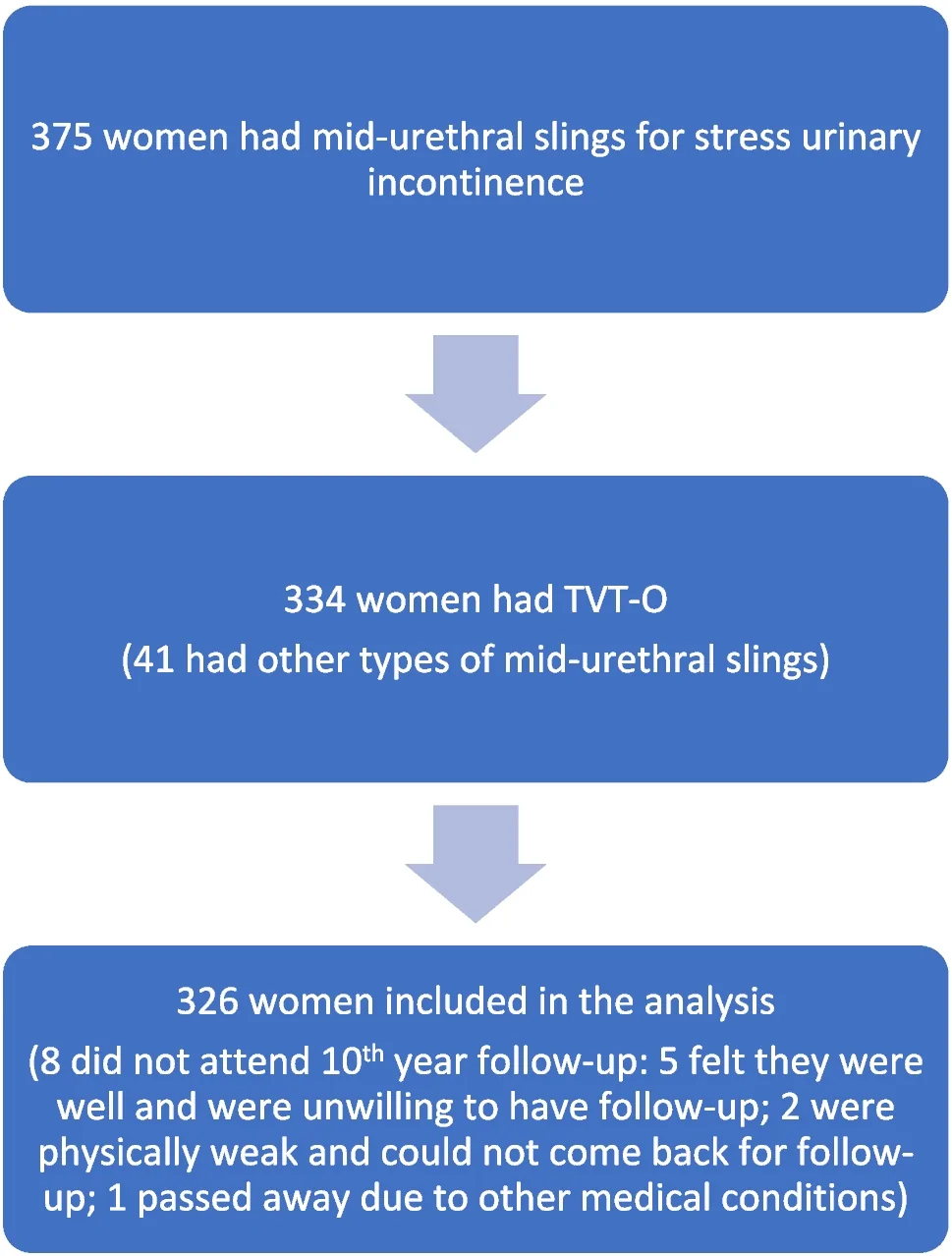
Flowchart of patient inclusion

During the period 2003–2015, a total of 375 women had a mid-urethral slings inserted for the treatment of SUI. Forty-one of them had mid-urethral slings other than TVT-O and were excluded from this study. Eight patients did not attend the follow-up at 10 years after TVT-O (Fig. 1). A total of 326 women were therefore included in this study. The mean age of the women at the time of surgery was 57.81 years (range 38–85 years). Other patient baseline characteristics are shown in Table 1.

**Table 1:** Baseline characteristics

Characteristics	Mean ± standard deviation (range)
Age at operation (years)	57.81 ± 9.96 (38–85)
Parity	2.68 ± 1.32 (0–8)
Number of vaginal deliveries	2.60 ± 1.36 (0–8)
Birth weight of heaviest baby (kg)	3.35 ± 0.50 (2–6)
Duration of stress urinary incontinence (years)	6.57 ± 5.86 (0–37)

Following the preoperative assessment, 62% (*n* = 202) of the women did not have POP. A total of 25.2% (*n* = 82) of the women had stage 2 POP and 43.9% (*n* = 143) of the women underwent concomitant surgery at the time of TVT-O. 85.3% (*n* = 122) of them had pelvic floor repair with or without hysterectomy, whereas 9.1% (*n* = 13) had mesh repair together with TVT-O.

Subjective and objective cure rates at 1, 5 and 10 years after surgery are summarised in Table 2. Both the subjective and objective cure rates were approximately 90% at 1 year after surgery and approximately 75% at 10 years after surgery.

**Table 2:** Subjective and objective cure rates at 1, 5 and 10 years after surgery

Outcomes	1 year	5 years		10 years	
	*N* (%)	*N* (%)	*p* value	*N* (%)	*p* value
Subjective cure rate	301 (92.3)	277 (85.0)		240 (73.6)	
		1st vs 5th year after surgery	< 0.001	1st vs 10th year after surgery 5th vs 10th year after surgery	< 0.001 < 0.001
Objective cure rate	298 (91.4)	279 (85.6)		245 (75.2)	
		1st vs 5th year after surgery	< 0.001	1st vs 10th year after surgery 5th vs 10th year after surgery	< 0.001 < 0.001

*N:* number

A total of 17.8% of the women had detrusor overactivity, as determined by the preoperative UDS (Table 3). This incidence was maintained at approximately 20% postoperatively, and there was no significant difference between the preoperative incidence and the postoperative incidence at 1, 5 and 10 years after surgery.

**Table 3:** Pre- and postoperative urgency urinary incontinence, detrusor overactivity and vaginal discomfort

Outcomes	Baseline	1 year		5 years		10 years	
	*N* (%)	*N* (%)	*p* value	*N* (%)	*p* value	*N* (%)	*p* value
Urgency urinary incontinence	204(62.6)	84 (25.8)		148 (45.4)		220 (67.5)	
		Preoperatively vs 1st year after surgery	<0.001	Preoperatively vs 5th year after surgery 1st vs 5th year after surgery	<0.001 <0.001	Preop Vs 10th year after surgery 1st Vs 10th year after surgery 5th Vs 10th year after surgery	0.18 <0.001 <0.001
Detrusor overactivity	58(17.8)	62 (19.0)		69 (21.2)		73 (22.4)	
		Preoperatively vs 1st year after surgery	0.71	Preoperatively vs 5th year after surgery 1st vs 5th year after surgery	0.16 0.33	Preop Vs 10th year after surgery 1st Vs 10th year after surgery 5th Vs 10th year after surgery	0.10 0.14 0.88
Vaginal discomfort	75(23.0)	15 (4.6)		13 (4.0)		22 (6.7)	
		Preoperatively vs 1st year after surgery	<0.001	Preoperatively vs 5th year after surgery 1st vs 5th year after surgery	<0.001 0.85	Preop Vs 10th year after surgery 1st Vs 10th year after surgery 5th Vs 10th year after surgery	<0.001 0.36 0.19

*N:* number

Three out of 326 patients reported thigh pain at 3 months after surgery, and none reported thigh pain at 10 years after surgery. Almost 1 in 4 women complained of vaginal discomfort before the operation, as assessed in the sixth question in the UDI-6 (Table 3). This percentage decreased to 4.6% at postoperative year 1, which was statistically significant. It increased to 6.7% at postoperative year 10, but this increase was not statistically significant compared with that at postoperative years 1 and 5, and the improvement in vaginal discomfort remained statistically significant compared with baseline.

A pad test was performed preoperatively and postoperatively. The weight of the pads significantly decreased from 28.0 ± 43.2 g preoperatively to 8.2 ± 23.9 g at postoperative year 10 (Table 4). Although the peak flow rate (PFR) slightly decreased after TVT-O, the PFR remained reasonably normal at postoperative year 10 (Table 4). There was a significant increase in postvoid residual urine volume postoperatively compared with baseline, but the absolute postvoid residual urine volume remained low (Table 4).

**Table 4:** Pre- and postoperative pad test, peak flow rate and postvoid residual urine volume results

	Baseline	1 year		5 years		10 years	
	Mean ± standard deviation	Mean ± standard deviation	*p *value	Mean ± standard deviation	*p* value	Mean ± standard deviation	*p* value
Pad test (g)	28.0 ± 43.2	4.2 ± 19.5		5.4 ± 20.6		8.2 ± 23.9	
		Preoperatively vs 1st year after surgery	<0.001	Preoperatively vs 5th year after surgery 1st Vs 5th year after surgery	<0.001 1.00	Preop Vs 10th year after surgery 1st Vs 10th year after surgery 5th Vs 10th year after surgery	<0.001 0.04 0.42
PFR (ml/s)	21.3 ± 7.8	17.5 ± 6.7		16.4 ± 6.3		15.1 ± 6.0	
		Preoperatively vs 1st year after surgery	<0.001	Preoperatively vs 5th year after surgery 1st Vs 5th year after	<0.001 <0.001	Preop Vs 10th year after surgery 1st Vs 10th year after surgery 5th Vs 10th year after surgery	<0.001 <0.001 <0.001
PVRU (ml)	3.1 ± 18.6	4.4 ± 16.9		8.5 ± 29.2		15.3 ± 30.6	
		Preoperatively vs 1st year after surgery	1.00	Preop Vs 5th year after surgery 1st Vs 5th year after surgery	0.03 0.13	Preop Vs 10th year after surgery 1st Vs 10th year after surgery 5th Vs 10th year after surgery	<0.001 <0.001 0.01

*PFR:* peak flow rate; *PVRU: *postvoid residual urine volume; *SD:* standard deviation

Quality of life was assessed using the validated UDI-6 questionnaire and the IIQ-7. The pre- and postoperative scores are listed in Table 5. The scores significantly improved after the operation. The mean VAS at the 10-year postoperative follow-up was 8.5 (range 5–10).

**Table 5:** Pre- and postoperative quality-of-life assessment using Urogenital Distress Inventory-6 (UDI-6) and Incontinence Impact Questionnaire-7 (IIQ-7)

	Baseline	1 year		5 years		10 years	
	Mean ± standard deviation	Mean ± standard deviation	*p* value	Mean ± standard deviation	*p* value	Mean ± standard deviation	*p* value
UDI-6	26.2 ± 14.2	3.2 ± 5.7		6.2 ± 8.0		11.1 ± 8.8	
		Preop Vs 1st year after surgery	<0.001	Preop Vs 5th year after surgery 1st Vs 5th year after surgery	<0.001 <0.001	Preop Vs 10th year after surgery 1st Vs 10th year after surgery 5th Vs 10th year after surgery	<0.001 <0.001 <0.001
IIQ-7	24.9 ± 24.1	1.1 ± 5.3		2.4 ± 9.2		3.7 ± 9.7	
		Preop Vs 1st year after surgery	<0.001	Preop Vs 5th year after surgery 1st Vs 5th year after surgery	<0.001 0.14	Preop Vs 10th year after surgery 1st Vs 10th year after surgery 5th Vs 10th year after surgery	<0.001 <0.001 0.34

*SD:* standard deviation

The postoperative complications are summarized in Table 6. Two women had tape erosion at the vagina, one of whom required excision of the exposed tape. Eight of the 81 patients (9.9%) diagnosed with recurrent USI underwent another mid-urethral sling procedure within 10 years of the original surgery. Therefore, the overall rate of repeating mid-urethral sling during the study period was 2.5% (8 out of 326).

**Table 6:** Postoperative complications

Complication	Number (%)
Bladder injury	1 (0.3)
Chest infection	2 (0.6)
Deep vein thrombosis	0 (0.0)
Fever	33 (10.1)
Urinary tract infection	5 (1.5)
Wound infection	1 (0.3)
Tape erosion	2 (0.6)

Subgroup analysis was performed on the subjective and objective cure rates at 10 years postoperatively between women who had TVT-O alone and women who had TVT-O plus concomitant surgery (Table 7). A total of 143 out of 326 women (43.9%) had concomitant surgery. 94.4% (135 out of 143) of concomitant surgeries performed were prolapse surgery. Both the subjective and objective cure rates at 10 years postoperatively were higher in women who had concomitant surgery but only the subjective cure rate reached statistical significance.

**Table 7:** Subgroup analysis at 10 years postoperatively between women who had transobturator mid-urethral sling (TVT-O) alone and women who had TVT-O plus concomitant surgery

	TVT-O alone, *n* = 183 (56.1%)	TVT-O plus concomitant surgery, *n* = 143 (43.9%)	*p* value
Age at operation, mean ± standard deviation	55.15 ± 9.15	61.22 ± 9.96	< 0.001
Parity, mean ± standard deviation	2.44 ± 1.28	3.00 ± 1.32	< 0.001
Subjective cure rate, *n* (%)	125 (68.3)	115 (80.4)	0.01
Objective cure rate,, *n* (%)	131 (71.6)	114 (79.7)	0.06

## Discussion

Our study reported the subjective and objective cure rates over a 10-year period in Chinese women who had TVT-O procedures for SUI. The default rate was 2.4%, which was low. Hong Kong is a small place and the Hospital is situated in the city centre. The health care cost for each follow-up is low. Women were informed of the follow-up arrangements when they entered the study. All of these facilitated compliance of our patients on their follow-up schedules.

A Cochrane review has affirmed the short-term and medium-term effectiveness and safety of mid-urethral sling operations, including transobturator and retropubic routes, as treatments for SUI in women [4]. However, long-term data regarding their effectiveness remain insufficient. Data regarding women with pure SUI treated with transobturator tension-free vaginal tape at 10 years, 13 years and 17 years of follow-up have been published from studies involving multiple centres in Switzerland, Italy and Greece [5, 7, 8]. The reported subjective cure rates were 97%, 95% and 81.4% at the 10-, 13- and 17-year follow-ups respectively. The objective cure rates, which were defined as stress tests at lithotomy and upright positions with a full bladder, were 92%, 92% and 80% at the 10-, 13- and 17-year follow-ups respectively. Our reported subject and objective cure rates were 73.6% and 75.2% respectively, 10 years after TVT-O, which were lower than the numbers quoted above. There are several reasons for this discrepancy. First, the hospital where this study was conducted is a urogynaecology subspecialty training centre. Therefore, almost all TVT-O procedures were performed by trainees under the supervision of experienced urogynaecologists. There is always a learning curve for a surgical procedure, including TVT-O. Second, the multi-centre group included only women with pure SUI and excluded those with mixed urinary incontinence and/or anatomical evidence of POP. Although the women included in the current study were heterogeneous, detrusor overactivity or overactive bladder symptoms and POP often co-exist with SUI, especially in the ageing population. Although the inclusion of these conditions may have an impact on the subjective and objective cure rates, these realistic numbers are important for preoperative patient counselling. Subgroup analysis has been performed on women with or without concomitant surgery. Women with concomitant surgery had a significantly higher subjective cure rate at 10 years but not objective cure rate. We postulated that this may be because these women had both their POP symptoms and their SUI symptoms cured; therefore, they felt subjectively better in all the aspects including SUI symptoms. However, as this was a subgroup analysis and the sample size in each arm is small, this observed result would probably need to be confirmed by a larger sample size. Third, we defined objective cure rates by repeating UDS at 1, 5 and 10 years after surgery rather than performing a stress test alone. This allows direct and strict comparisons with the baseline values. Fourth, we included 326 women who completed 10 years of follow-up, which was double the number in the study by Serati et al. [7]. Despite the lower subjective and objective cure rates, there were significant improvements in the TVT-O of patients, as determined using the UDI-6 questionnaire, IIQ-7, pad test and VAS.

De novo overactive bladder symptoms are known complications after mid-urethral sling operations. The prevalence rates were 14%, 16% and 18.5% at the 10-year, 13-year and 17-year follow-ups respectively, according to the studies by Serati et al. [5, 7, 8]. It is difficult to calculate this rate from our study, as we included women with preexisting mixed urinary incontinence and detrusor overactivity. However, there was no statistically significant increase in detrusor overactivity rates at 1, 5 and 10 years after surgery or at baseline.

In the ESTER systematic review, the rate of tape and mesh exposure was higher after TVT-O procedures than after retropubic mid-urethral slings [9]. Only 2 women (0.6%) experienced tape erosion within 10 years after surgery in the current study, and only 1 of them required local excision for tape erosion. Similarly, serial studies by Serati et al. [5, 7, 8] reported low tape erosion rates of 0% at 10 years, 2.5% at 13 years and 10% at 17 years.

Thigh pain and vaginal discomfort after TVT-O operations have been a concern for many urogynaecologists and patients. Our study revealed no thigh pain at the 10-year follow-up assessment and a statistically significant improvement in vaginal discomfort after TVT-O, which was maintained at the 10-year follow-up assessment. These favourable results echo those of other studies regarding the long-term outcomes of TVT-O [5, 7, 8].

The rates of repeating mid-urethral sling for recurrent SUI were 5.2% at 9 years and 1.6% at 8 years in the studies by Berger et al. [10] and Mengerink et al. [11] respectively. Our reoperation rate of 2.5% at 10 years is in line with that mentioned in the literature.

One strength of this study is the large sample size of 326 women who completed 10 years of follow-up. Another important feature of this study is that it focuses on Chinese women, which constitute the majority of the ethnicity in our local population. The results of this study are therefore particularly useful when counselling the Chinese population.

Although women with mixed urinary incontinence/detrusor overactivity and POP were included in this study, which may reduce the subjective and objective cure rates, it is important to conduct studies with a study population similar to the real population. Overactive bladder symptoms/detrusor overactivity and POP commonly co-exist with SUI. Therefore, the results of this study provide our women with realistic counselling information. Another limitation of this study is the lack of generalisability of the results to the non-Chinese population as only Chinese women were included in this study.

## Conclusion

The TVT-O is a highly effective and safe surgical treatment for women with SUI based on results obtained 10 years after surgery. Thigh pain after TVT-O is rare and should not prevent women from receiving this useful treatment.
